# Haplotype variations of sucrose phosphate synthase B gene among sugarcane accessions with different sucrose content

**DOI:** 10.1186/s12864-023-09139-1

**Published:** 2023-01-25

**Authors:** Hongbo Liu, Xiuqin Lin, Xujuan Li, Ziliang Luo, Xin Lu, Qian You, Xiping Yang, Chaohua Xu, Xinlong Liu, Jiayong Liu, Caiwen Wu, Jianping Wang

**Affiliations:** 1 Sugarcane Research Institute, Yunnan Academy of Agricultural Sciences/Yunnan Key Laboratory of Sugarcane Genetic Improvement, Kaiyuan, Yunnan 661699 China; 2 Agronomy Department, University of Florida, Gainesville, FL 32610 USA; 3 Genetics Institute, Plant Molecular and Biology Program, University of Florida, Gainesville, FL 32610 USA; 4 Guangxi Key Laboratory for Sugarcane Biology, Guangxi University, Guangxi 530004 Kaiyuan, China

**Keywords:** Sugarcane, Sucrose phosphate synthase B (*SPS*B) gene, Haplotype, Sucrose

## Abstract

**Background:**

Sucrose phosphate synthase B (*SPS*B) gene encoding an important rate-limiting enzyme for sucrose synthesis in sugarcane is mainly expressed on leaves, where its alleles control sucrose synthesis. In this study, genetic variation of *SPS*B gene represented by different haplotypes in sugarcane was investigated in hybrid clones with high and low sugar content and various accessory species.

**Results:**

A total of 39 haplotypes of *SPS*B gene with 2, 824 bp in size were identified from 18 sugarcane accessions. These haplotypes mainly distributed on Chr3B, Chr3C, and Chr3D according to the AP85-441 reference genome. Single nucleotide polymorphisms (SNPs) and insertion/deletion (InDels) were very dense (42 bp/sequence variation) including 44 transitional and 23 transversional SNPs among the 39 haplotypes. The sequence diversity related Hd, Eta, and Pi values were all lower in clones of high sucrose content (HS) than those in clones of low sucrose content (LS). The evolutionary network analysis showed that about half *SPS*B haplotypes (19 out of 39) were clustered into one group, including 6 (HAP4, HAP6, HAP7, HAP9, HAP17 and HAP20) haplotypes under positive selection in comparison to HAP26 identified in Badila (S. *officinarum*), an ancestry noble cane species and under purification selection (except HAP19 under neutral selection) in comparison to HAP18 identified from India1 (S. *spontaneum*), an ancestry species with low sugar content but high stress tolerance. The average number of haplotypes under positive selection in HS clones was twice as that in LS. Most of the SNPs and InDels sequence variation sites were positively correlated with sucrose and fiber content and negatively correlated with reducing sugar.

**Conclusions:**

A total of 39 haplotypes of *SPS*B gene were identified in this study. Haplotypes potentially associated with high sucrose synthesis efficiency were identified. The mutations of *SPS*B haplotypes in HS were favorable and tended to be selected and fixed. The results of this study are informative and beneficial to the molecular assisted breeding of sucrose synthesis in sugarcane in the future.

**Supplementary Information:**

The online version contains supplementary material available at 10.1186/s12864-023-09139-1.

## Background

Sucrose plays a key role in plant growth and development. It is mainly synthesized in the cytoplasm of leave cells. Carbon dioxide (CO_2_) from the air enters the leaves of plants and is fixed into triose phosphates. Most of the triose phosphate generated is converted to sucrose, which starts from condensation of two triose phosphates to form a fructose 1, 6 biphosphate, and then is hydrolyzed to yield fructose 6 phosphate. Sucrose 6-phosphate synthase catalyzes the reaction of fructose 6-phosphate with uridine diphosphate glucose (UDPG) to form sucrose 6-phosphate. Finally, sucrose 6-phosphate phosphatase removes the phosphate group of sucrose 6-phosphate making sucrose available to be transported to other places of the plants as energy or for storage. Sucrose phosphate synthase (SPS) is a key and irreversible rate-limiting enzyme of sucrose synthesis in plants by catalyzing the conversion of fructose 6-phosphate and UDP-glucose into sucrose-6-phosphate, the substrate for a final step of sucrose synthesis.

Sugarcane is an important economical crop primarily for sugar production. Its sucrose yield can reach up to 50% of dry weight [1]. Extensive studies have been conducted to investigate the gene pathways involved in sucrose synthesis, transportation, and storage [2–9]. Beside the sucrose phosphate synthase genes, the invertase family genes, sucrose synthase family genes, sucrose transporter family genes (*SUTs*) also have important influence on sucrose accumulation. Sucrose synthase can not only promote the synthesis of sucrose, but also promote the decomposition of sucrose. Sucrose invertase can promote the conversion of sucrose into fructose and starch. Sucrose transporter transport sucrose from the original source to the sink tissue. However, due to the complexity of sugarcane genome, the progress has been slow. Sugarcane is highly polyploidy with chromosome number ranged between 80 and 130 in a ploidy level up to 13x [10]. Therefore, at every single gene locus, multiple alleles on different homo(eo)logous chromosomes (up to 13) are exist, which interact together with certain dosage effects to determine the gene function, no mention about the paralogs in the gigantic genomes of sugarcane.

As a critical enzyme in sucrose synthesis, the SPS is encoded by *SPS* gene, which has five groups in sugarcane genomes, named *SPS*A, *SPS*B, *SPS*C, *SPS*D1 and *SPS*D2 [11, 12]. The expression patterns of the five *SPS* genes in different sugarcane tissues were different [13]. The *SPS*C predominantly expressed in both immature and mature leaves [14]; whereas *SPS*D expressed at similar levels in all tissues examined; *SPS*A has low expression in leaves and gradually increasing expression level from the meristem region down to internode 7 of the stem [15]. Except *SPS*A, all *SPS* genes expressed significantly higher in accessions with high sucrose content than in accessions with low sucrose content [13, 16]. Linkage analysis of a sugarcane mapping population revealed that *SPS* genes located in different linkage groups and *SPS*D genes were strongly associated with sugar-related traits [17]. The SPS activity and sucrose content were both enhanced in sugarcane with *SPSA* gene overexpressed, in addition to increased plant height and effective stem number [18], consistent with McIntyre's findings [17]. The *SPS*B predominantly expressed in both immature and mature leaves with the highest expression in the whole *SPS* family genes and was considered to be positively correlated with sucrose content [14]. Improving sucrose content has been one of the major ultimate goals of sugarcane breeding programs. In traditional sugarcane breeding programs, several desirable traits are targeted for selection, which are influenced by allelic (intragenic) interaction, intergenic interaction, environmental factors and interactions between genetic and environmental factors, making the precise selection extremely challenging. Dissecting the effects of different genes, alleles, and environmental factors on the traits of interest are critical for effective selection in the breeding programs. Identifying different gene haplotypes and their functions are one of the important steps in dissecting the genetic factors controlling agronomic traits.

However, there were few reports on the haplotype study of functional genes in sugarcane. Previously haplotypes of sucrose transporter genes, sucrose synthase genes were identified and compared by sequencing homologous bacterial artificial chromosomes (BACs) and further approved that every *SPS* gene does have many haplotypes [9, 19]. However, which haplotypes contribute to sucrose accumulation in sugarcane were largely unknown. Sucrose is mainly synthesized in the leaves. Of the whole *SPS* family genes, *SPS*B had a high expression in both immature and mature leaves and was considered to be positively correlated with sucrose content particularly at the early stage of stem sucrose accumulation [15]. This current study is aimed to investigate the genetic variation of different haplotypes or alleles of *SPS*B gene in different sugarcane accessions with contrastive sucrose content to identify the haplotypes potentially associated with sucrose content. The results of this study will provide valuable reference for haplotype selection in sugarcane to enable molecule assisted breeding for sucrose content improvement.

## Results

### Sucrose content analyses

According to the Brix measured in the field, a total of 6 high sucrose (HS) hybrid clones including YZ02-588, DeZhe93-88, YT00-236, YZ14-401, YZ14-405, YZ14-407, and 7 low sucrose (LS) hybrid clones: GT12, YZ94-343, YZ14-402, YZ14-403, YZ14-404, YZ14-406 and YZ14-408 were classified (Table 1). The Brix difference of all the accessions ranged from 1.93 to 7.93. The average sucrose content in HS group was 15.90%, higher than that in LS, 13.04%, also higher than that of ROC22 (13.07%), and Badila (*Saccharum officinarum*) (15.2%). The ANOVA analysis showed that there were significant differences (*P* < 0.01) between HS and LS groups in terms of Brix (%), sucrose content, and reduced sucrose, but no significant differences within the group (Table 1).

**Table 1:** The ANOVA results between or within groups of high sucrose content and low sucrose content

Characters	Group	Sq	df	Mse	F value	Significance(*P-*value)
Brix	Between group	54.139	1	54.139	40.257	0.00^**^
	Within group	14.793	11	1.345	_	_
Sucrose content	Between group	26.374	1	26.374	10.964	0.007^**^
	Within group	26.46	11	2.405	_	_
Reducing sucrose	Between group	1.603	1	1.603	20.493	0.001^**^
	Within group	0.86	11	0.078	_	_

^**^ Significance different at *P* < 0.01. *Sq* chi-square, *df* degrees of freedom, *Mse* Mean Squared Error

### The amplification of *SPSB* gene and sequence analysis

The *SPS*B gene was successfully amplified from 18 accessions with an amplificon size of 2,824 bp (Additional file 1). A total of 1080 amplicon clones (60 clones for each accession’s amplicon) were picked for Sanger sequencing. In total, 869 amplicon clones were successfully sequenced and 567 of them were assembled into contigs. The number of assemblies derived from each accession ranged from 7 to 52. Through sequence alignment and comparison, a total of 39 haplotypes were identified from the 567 assemblies (Table 2, NCBI accessions: OP615365 ~ OP615403). In all of *SPS*B haplotypes, the sugarcane ancestral species, *S. officinarum* (Badila) and *S. spontaneum* (India1) each contained only one haplotype, while the ancient sugarcane variety, Guangze bamboo cane (*S. sinense*) possessed four haplotypes, and Katha (*S. barberi*) had two haplotypes. In the hybrid clones, the number of identified haplotypes ranged from 1 to 13. The 6 HS and 7 LS clones had a total of 60 and 56 haplotypes, respectively, including 9 HS-specific and 5 LS-specific haplotypes, respectively. The haplotype HAP16 only existed in Guangze bamboo cane. All of the haplotypes were located on Chr3D, Chr3C, and Chr3B of *S. Spontaneum* genome (http://www.life.illinois.edu/ming/downloads/Spontaneum_genome) (Additional file 2). Most haplotypes (38) were highly homologous compared with Chr3D (99% with no or less gap), Chr3C (98–99% with a few gaps), Chr3B (98–99% with a lot of gaps), except Hap17 has highly homologous with Chr3B. Four haplotypes (HAP6, HAP25, HAP33 and HAP38) showed partial homology on other chromosomes of *S. spontaneum* genome. Among these haplotypes, 18 were caused by intron mutations and 21 were caused by exon mutations. Further look into the partial homology revealed that HAP6 had a Harbinger transposon (Targer site Duplication was "TAA") inserted into the 7th intron of *SPS*B gene (Predict URL: https://www.girinst.org/) and HAP25, HAP33 and HAP38 had 277 bases inserted in the third intron of the *SPS*B gene [20].

**Table 2:**
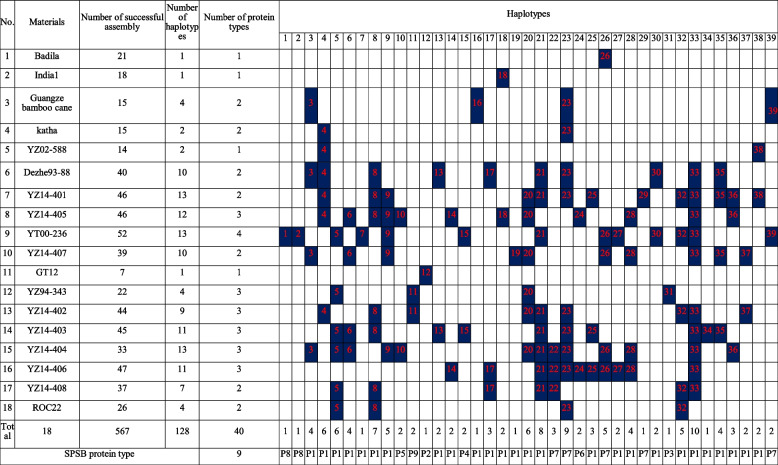
The haplotype distribution of SPSB gene in 18 accessions

The blue highlighted and numbers represent the identified haplotypes of the accession

### Genetic variation analysis of *SPSB* alleles

Among the 39 haplotypes, 67 SNPs and 6 InDels were identified with an average SNP density of 42 bp/SNP. Transition SNPs occurred at 44 sites, and transversion at 23 sites in the 39 *SPS*B haplotypes (Additional file 3). The haplotype diversity (Hd), total number of mutations (Eta), number of polymorphic (segregating) sites (S), and variance of haplotype diversity (Hv) in HS clones were all relatively lower than those in LS group (Table 3), which indicated that the mutations in HS were favorable and tended to be selected and fixed.

**Table 3:** The genetic diversity analysis of haplotypes from high sucrose clones and low sucrose clones

Items	Total accessions	HS	LS
Number of accessions	18	6	7
Number of haplotype (Hn)	39	33	29
Total numberer of mutations (Eta)	68	53	57
Number of polymorphic(segregating) sites (S)	66	53	56
Haplotype (gene) diversity (Hd)	0.999	0.9757	0.998
Variance of haplotype diversity (Hv)	0.00004	0.00007	0.00009
Nucleotide diversity (Pi)	0.00287	0.00268	0.00274

HS means high sucrose content clones, LS means low sucrose content clones

**Fig. 1:**
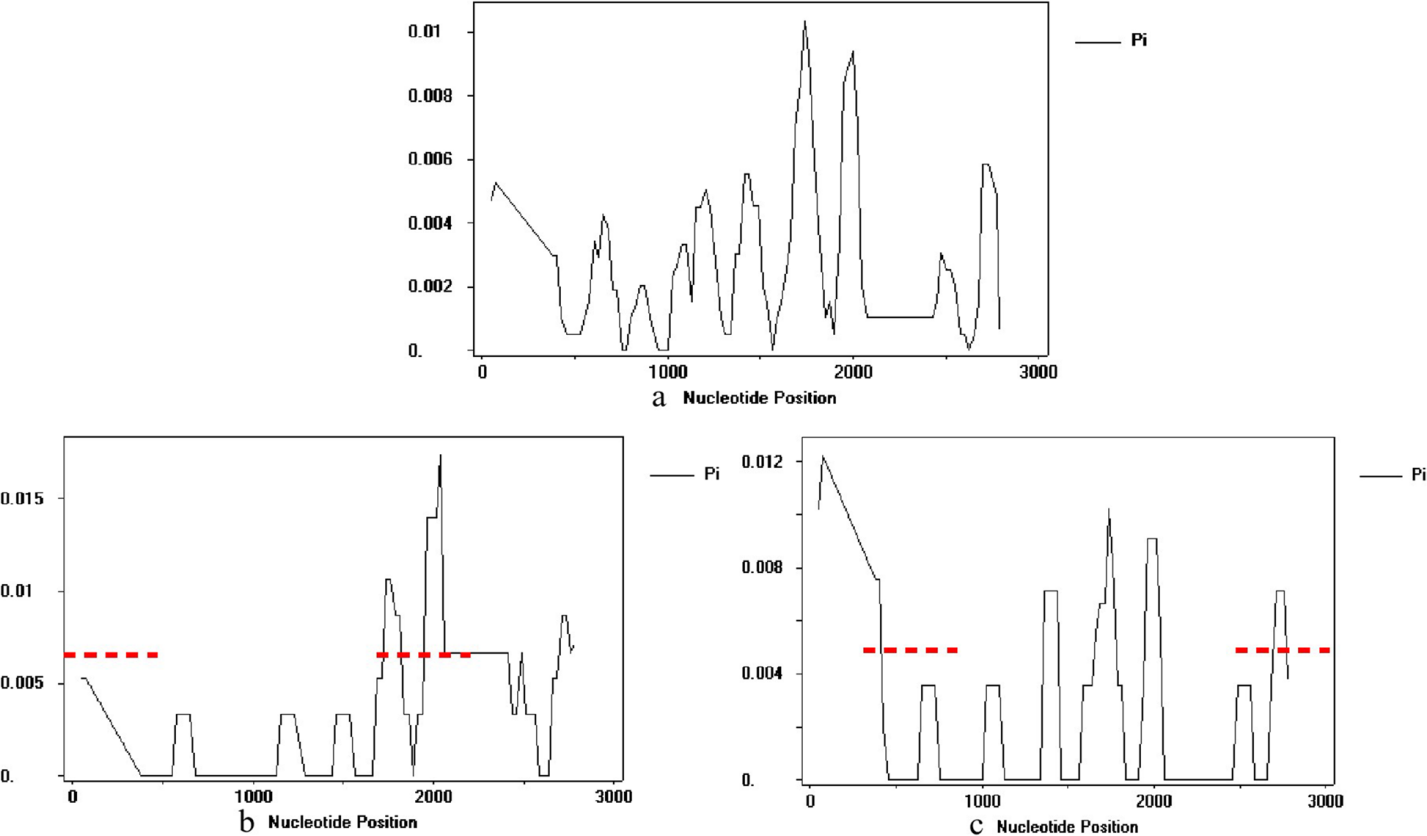
Nucleotide variations along the SPSB gene among the (**a**) 39 haplotypes, (**b**) haplotypes of low sucrose content clones, and (**c**) haplotypes of high sucrose content clones. The ordinate represented the nucleotide diversity(Pi), the red dot represented the area of difference

The nucleotide diversity (Pi) of 39 *SPS*B haplotypes showed that the nucleotide mutation positions scattered across the *SPS*B gene (Fig. 1a). The haplotypes in HS groups had much higher variations in the first 500 bp of the *SPS*B amplicon sequences while the haplotypes in LS groups had high variation at 2000–2500 bp region (Fig. 1b, c). The high variation in HS contained the SNP mutations in haplotypes, HAP1, HAP5, HAP7, and insert sequence in HAP25, HAP33, and HAP38. The average Pi value of HS was 0.00268, lower than that of LS (0.00275) (Table 3), indicating that the DNA sequences in HS group was relatively conservative comparing to LS. Through variable splicing enzyme recognition site analysis, HAP25, HAP33 and HAP38 had YNYYRAY-type recognition sites [21] in the 3^rd^ intron, and HAP6 had two and one YNYYRAY-type recognition sites in the 7^th^ and 8^th^ intron, respectively. Compared with other haplotypes, HAP15 had an additional YNYYRAY recognition site in the 4^th^ intron. According to these haplotype variable splicing recognition sites, there was no significant difference in the distribution of HAP6, HAP15, HAP25, HAP33, and HAP38 between the HS and LS groups.

With ROC22 *SPS*B cDNA (ID: JN584485) as a reference, the coding regions of 39 *SPS*B haplotypes encoded nine different protein types, of which HS contained six and LS contained eight. A total of 26 haplotypes encoded one protein type (P1 type, Table 2). Majority of the accessions (75%) had these 26 haplotypes. Particularly, HS had an average of 8.7 P1 type haplotypes, while LS had 5.7 P1 type haplotypes. Protein type P7 was the second popular protein type in all the accessions, with 15% of the total haplotypes encoding this protein type.

### Haplotype network and phylogenetic analysis

**Fig. 2:**
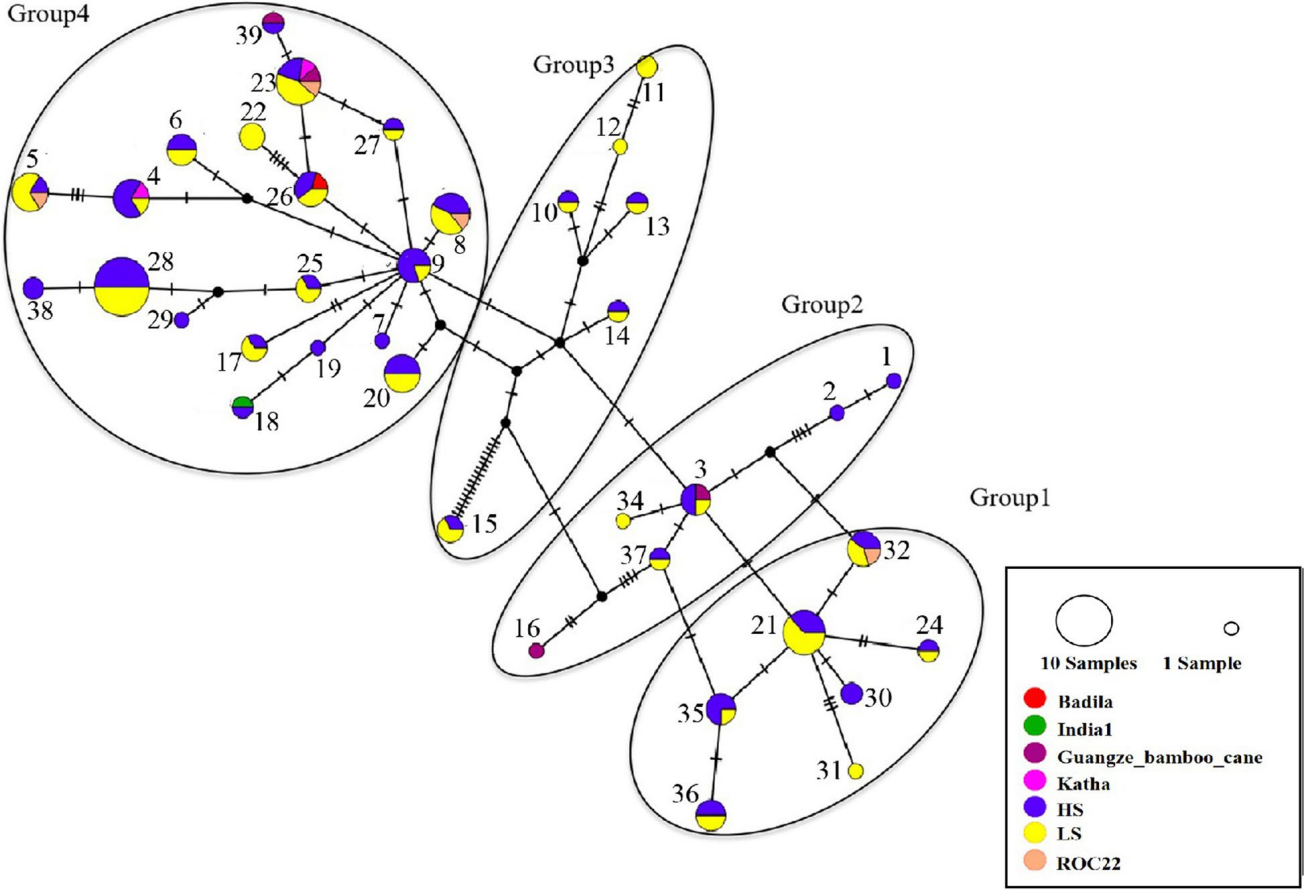
The evolutionary network for the 39 haplotypes. Each link between haplotypes (presented in numbers) represents the minimum of mutational difference. Unlabeled nodes indicate inferred steps not found in the sampled populations. One transversal represents one SNP. The circle size represents the frequency of haplotypes in 18 accessions included in this study

According to the characteristics of haplotype evolutionary network relationship, the haplotypes were divided into four groups (Fig. 2). In Group 1 there were 7 haplotypes encoding 3 protein types, Group 2, 6 haplotypes encoding 2 protein types, Group 3, 6 haplotypes encoding 5 protein types, and Group 4, 19 haplotypes encoding 2 protein types, P1 and P7, the main protein types in all accessions. HAP9 was distributed in the center of Group 4, thus was considered as the primitive haplotype. In addition, the haplotypes of HAP18, HAP26, HAP23 and HAP39 derived from the four ancestral *Saccharum* clones, India1 (*Saccharum spontaneum*), Badila (*Saccharum officinarum*), Katha (*Saccharum barberi* J), and Guangze bamboo cane (*Saccharum sinense* R.), respectively, were all clustered in the group 4 centered with HAP9, which further indicated that other haplotypes in Group 4 may have evolved from HAP9. In HS clones, the six HS-specific haplotypes were all clustered in Group 4; while the five LS-specific haplotypes mostly scattered into other different groups, specifically, HAP11 and HAP12 in Group 3, HAP31 in Group 1, HAP34 in Group 2, and only HAP22 in Group 4 at a distal area.

### Analysis of evolutionary selection effect of *SPSB* haplotypes

**Fig. 3:**
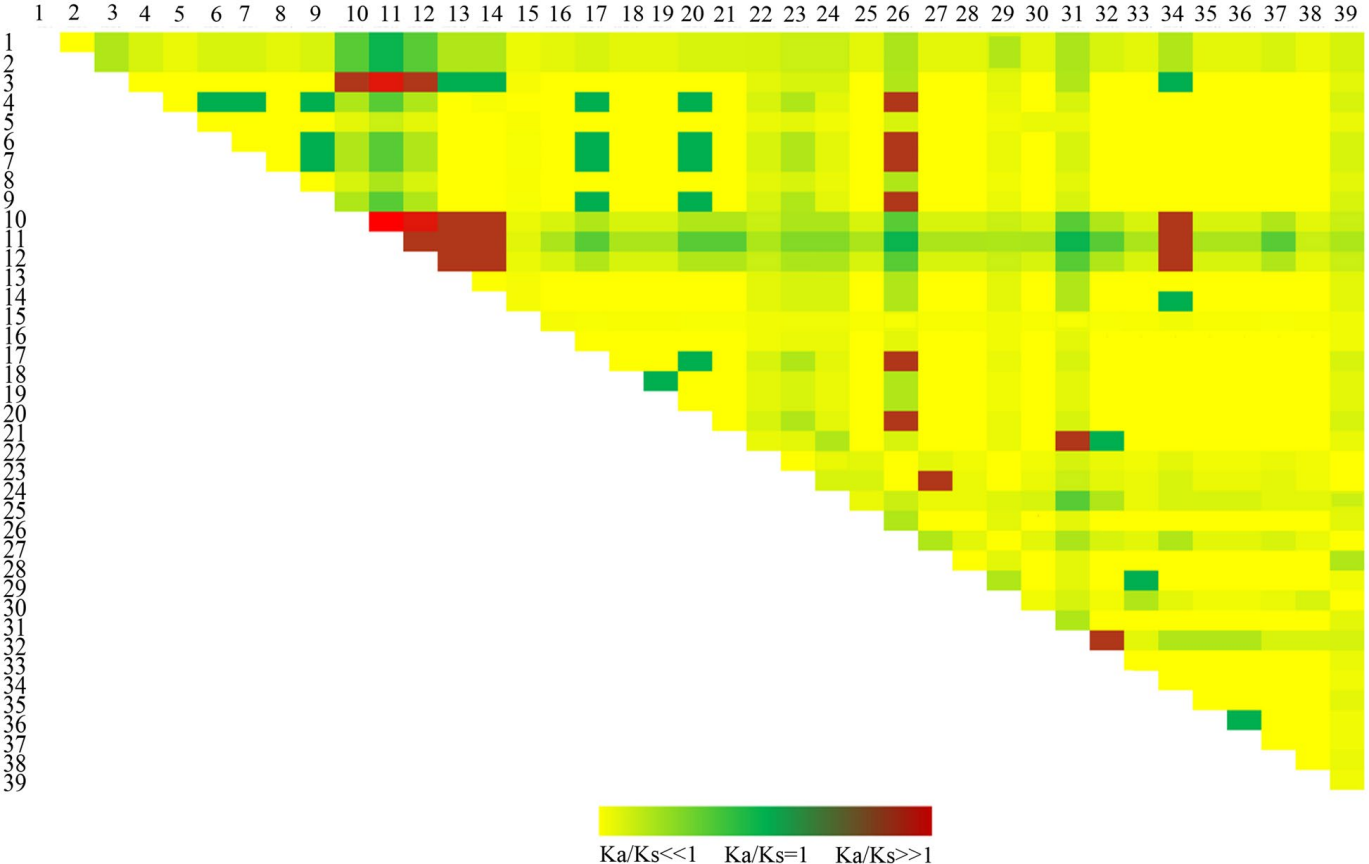
The heatmap of Ka/Ks of the 39 haplotypes

The analysis of synonymic and non-synonymic mutations between haplotypes showed that the 39 *SPS*B haplotypes were either under positive, neutral, or purification selection (Fig. 3). With the HAP26 haplotype of Badila (*S. officinarum*) as the reference, only six (HAP4, HAP6, HAP7, HAP9, HAP17, and HAP20) of 39 haplotypes were under positive selection, all encoding protein 1 and were all in Group 4 in the TCS network (Fig. 2). Interestingly, the average number of the haplotypes under positive selection was 2 in HS group (Additional file 4), which was more than that (1) in LS group. In HS (DZ93-88, YZ14-401, YZ14-405, YZ14-407 and YT00-236), each of the accession contained two or more haplotypes under positive selection. While as in LS group, except YZ14-402 contained two haplotypes under positive selection, all other accessions contained only 1 or 0 haplotypes under positive selection. Particularly, HAP7, a HS-specific haplotype, was one of those under positive selection. HAP22, the only LS-specific haplotype in Group 4 in the TCS network was under purification selection.

### Correlation analysis of different *SPSB* haplotypes and sugar traits

**Fig. 4:**
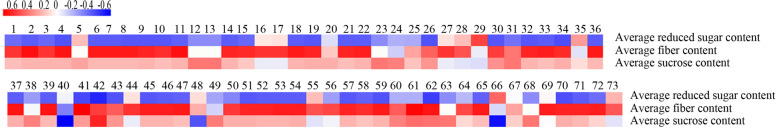
Sequence variations among 39 SPSB gene haplotypes and their correlations with sucrose content, fiber content and reduced sugar content. Notes: number 1–73 on the top row indicate SNP and InDels sites. Dark red box represents an extremely significant positive correlation. Dark blue represents an extremely significant negative correlation

Correlation analyses between sucrose, fiber, reducing sucrose and nucleotide sites with variation among *SPS*B haplotypes were conducted, respectively, with the HAP26 haplotype of *SPS*B gene of Badila as the reference (Fig. 4). Among the SNP and InDels mutation sites (a total of 73) of 39 haplotypes of *SPS*B gene, most of the 73 variation sites were positively correlated with sucrose content and fiber content, but negatively correlated with reducing sugar content. However, several mutation sites such as the 29^th^ nucleotide of HAP1, 2, 40^th^ of HAP16, 48^th^ of HAP10, 11,12,13, and 66^th^ of HAP22 were negatively correlated with sucrose content and positively correlated with reduced sugar content. In the sibling sugarcane hybrid clone pairs with contrastive sugar content, the sucrose content of YZ14-407 without HAP22 was higher than that of YZ14-406 with HAP22, the same as the sibling pair of YZ14-405 and YZ14-404. The haplotypes containing the nucleotide sites correlated to high sucrose and fiber contents and low reduced sugar content, which presumably will be selected in the breeding programs are 31^st^ (HAP4, HAP5), 42^nd^ (HAP1, HAP2, HAP3, HAP21, HAP24, HAP30, HAP31, HAP32, HAP34, HAP35, HAP36, HAP37), 60^th^ (HAP8), and 61^th^ (HAP6) nucleotide sites, among which, 4.6 haplotypes on average were in HS group, and 3.2 in the LS. These haplotypes will be further investigated for their potential for increasing sucrose content in sugarcane.

## Discusion

The study of gene haplotypes provides details of genetic variation gene sequences, which allow us to deeply understand the historical characteristics of gene domestication. The results lay foundations for accelerating functional genome and molecular breeding research in sugarcane particularly with its high polypoid nature. Genetic variation in different haplotypes of functional genes can affect phenotypic or agronomic traits in plants, such as those found in studies of soybean *GmST05* gene haplotypes, the natural variation in *GmST05* determines transcription levels and influences seed size and quality in soybean [22]. In sugarcane, *SPS* gene plays a critical role in controlling sucrose synthesis and different haplotypes play different functions in sugarcane and add another layer to the function complexity of gene expression related to sucrose synthesis [23, 24]. Accurate identification of different *SPS*B haplotypes in sugarcane genomes are critical to understand the genetic control of the sucrose synthesis process, sucrose content difference between different accessions, which will provide referable information on sucrose content improvement in sugarcane breeding programs.

### Reliability analysis of *SPSB* gene haplotype

Identifying gene haplotypes relies on high fidelity during gene fragment amplification, cloning, and sequencing to capture every possible and biological sequence variation among the haplotypes with no or minimum sequence errors. In our study, high fidelity KOD polymerase, which has a very low mutation frequency (0.09%), was used. Meanwhile, every single amplicon of 2,824 bp *SPS*B gene fragment was cloned, and 60 independent clones were picked and sequenced to capture all the possible haplotypes within each amplicon of the target gene. Extensive manual checks were conducted to ensure the reliability of the reads with no ambiguous bases allowed. Meanwhile, only the haplotype with more than two identical assemblies were considered as a true and reliable haplotype. Of the 39 *SPS*B haplotypes identified, almost every haplotype had mutations at multiple sites, which were also presented in more than one accession, which further validate the reliability of the haplotype calling. However, due to the high polyploid level of sugarcane, it is possible that we missed detecting all the possible haplotypes, particularly the rare alleles from certain accession. Statistically, we may need 56 successful assemblies from each accession (according to binomial distribution) to not miss any minor haplotype for a 12 × polyploid of sugarcane [25]. However, in our study, we generated 31.5 assemblies per accession in average, ranged from 7 to 52 assemblies across the 18 sugarcane accessions. Some minor haplotypes might not be detected due to low number of successful assemblies. The 39 *SPS*B haplotypes identified in this study should represent the most dominant haplotypes. Majority of the haplotypes appeared multiple times in different accessions. The most sugarcane accessions had more than one haplotype and some had high number of haplotypes closing to their ploidy level. The downstream genetic variation and correlation analyses of these dominant haplotypes should be informative and valuable for our research goal.

### Genetic variation analysis of different haplotypes of *SPSB* gene

The 39 *SPS*B gene haplotypes were highly variable with not only frequent SNPs, but also some InDels. For example, HAP15 had a 6-bp insertion in the 4^th^ intron and formed inverted repeats with the existing intron sequence. In HAP17 sequence, a 11-bp was missing from the 7^th^ intron and a 19-bp was inserted in the 4^th^ intron as a direct repeat sequence with its downstream sequence. Due to transposon insertion, HAP6 may be de-methylated to achieve higher expression efficiency [22]. The insertion sequence in HAP25, HAP33 and HAP38 need to be further validated for their relationship with sucrose content. Among the haplotypes produced by intron mutation, HAP2 and HAP1 has a single base mutation in the 3^rd^ intron, HAP7 has a mutation of T-C base in the 4^th^ intron, resulting in its separation from several other haplotypes. The sequence variation in introns among haplotypes could possibly affect the alternative splicing, thus the structure and function of SPSB proteins [26]. In the TCS network analysis, Group 4 was considered as a cluster of haplotypes under positive selection, which also contained haplotypes from ancestral accessions of *S. officinarum*, *S. spontaneum*, *S.sinense* and *S. barberi*. The results suggested that the haplotypes under positive selection were most likely evolved recently from the ancestral accessions and are contributing to sucrose accumulation in modern sugarcane cultivars. In this study, three pairs of sister lines from the same cross showing contrastive sugar content were compared. Between the pair of sister clones, YZ14-401 and YZ14-402, the HAP9 under positive selection was only present in the high sugar genotype YZ14-401 but not in the low sugar genotype, YZ14-402. The HAP38 from Group4 also exists only in YZ14-401. The results suggested that the Group 4, which contained *SPS*B haplotypes under positive selection may contribute to a high efficiency in sucrose synthesis. The sister pair clones, YZ14-406 and YZ14-407 had 11 (encoding 3 protein types) and 10 (encoding 2 protein types) haplotypes, respectively, with 3 of them as common ones. The high sugar clone, YZ14-407, had 3 haplotypes under positive selection and the low sugar line, YZ14-406 only had 1 under positive selection. Between sister clones YZ14-404 and YZ14-405, there were 7 common haplotypes. Again, HAP4 under positive selection only existed in high sugar clone. These results indicated that the haplotype under positive selection had important effects on the sucrose synthesis. Meanwhile, dose effect appeared to contribute to the sucrose content. It was noticed that the number of haplotypes under positive selection in high sucrose content clones was higher than that in low sucrose content clones.

Most SNPs and InDels among the 39 haplotypes were positively correlated with sucrose content and fiber content in sugarcane, but negatively correlated with reduced sugar content, which was most likely the results of the long-term domestication and artificial selection of high sugar content of sugarcane clones. Those drastic nucleotide changes such as transversion instead of transition SNPs or big insertion and deletions can change the functionality of *SPS*B, which may be beneficial to sucrose synthesis and accumulation and thus were selected during domestication and breeding processes.

## Conclusions

*SPS*B gene showed extensive sequence variations in the sugarcane accessions in this study with a huge number of haplotypes or alleles. The number of *SPS*B haplotype under positive selection was more in HS clones than that in LS clones. The Hd, Eta, and Pi values were all lower in HS clones than those in LS clones. The evolutionary network of all the haplotypes demonstrated that *SPS*B haplotypes in Group 4 may derived from the haplotype of Badila, an old ancestry noble cane with high sucrose content. The group 4 also contained all haplotypes (HAP4, 6, 7, 9, 17, 20) under positive selection and demonstrating dosage effect. The SNPs and InDels of *SPS*B gene haplotype under positive selection were correlated with sucrose content and fiber content. The results in this study laid the foundation for further analysis of the functional alleles contributing to sucrose accumulation, which will allow us to develop the functional markers to assist selection of breeding accessions for sugarcane cultivar improvement.

## Methods

### Plant materials

A total of 18 *Saccharum* accessions were used in this study, including 4 *Saccharum* ancestry accessions: Badila, India1, Guangze bamboo cane, and Katha, belonging to S. *officinarum* L., S. *spontaneum* L., S. *sinense* R., and S. *barberi* J., respectively, 6 commercial sugarcane varieties with high or low sucrose performance in commercial production, and 8 interspecific hybrid clones from 5 different cross combinations (Table 4), among which, three pairs of lines with contrastive sucrose content according to Brix (%) in the field were selected, YZ14-401 and YZ14-402, YZ14-404 and YZ14-405, YZ14-406 and YZ14-407 with each pair derived from the same cross. The 4 *Saccharum* ancestry accessions and 7 commercial varieties were provided by National Nursery of Sugarcane Germplasm Resources (NNSGR) in Kaiyuan, Yunnan province of China; the 8 hybrid lines were provided by Sugarcane Research Institute, Yunnan Academy of Agricultural Sciences, Yunnan province of China (YAAS, YSRI). All the above accessions were planted in the first field station of YSRI in January 2017. Each accession was planted in 3 4-m trenches under maintenance in the same condition as the commercial sugarcane cultivars.

**Table 4:** Sugar measurement of all the accessions used in this study

Characteristic	Accession	Species name or feature	Parents of hybrids	Field Brix%(Dec)	Average sucrose content%	Average fiber content%	Average reduced sugar content%
HS	YZ02-588	Hybrid cultivar	CP72-1210 × YT84-3	23.6	16.12	11.34	0.24
	YZ14-401	Hybrid breeding line	YC07-71 × HoCP01-517	23.21	15.86	14.27	0.49
	YZ14-405	Hybrid breeding line	Co1001 × ROC25	23.71	15.86	16.25	0.27
	YT00-236	Hybrid cultivar	YN73-204 × CP72-1210	22.25	16.26	11.35	0.34
	Dezhe93-88	Hybrid cultivar	YC71-374 × CP72-1210	22.44	15.54	14.07	0.34
	YZ14-407	Hybrid breeding line	ROC10 × YT93-159	22.34	15.76	15.08	0.78
LS	YZ94-343	Hybrid cultivar	ROC10 × YC82-96	18.9	14.85	11.28	1.27
	GT12	Hybrid cultivar	CO419 × CZ57-416	19.41	13.87	11.89	1.5
	YZ14-406	Hybrid breeding line	ROC10 × YT93-159	20.32	13.83	13.2	0.67
	YZ14-403	Hybrid breeding line	YZ94-343 × ROC22	19.13	13.2	15.56	1.01
	YZ14-404	Hybrid breeding line	Co1001 × ROC25	19.62	13.99	13.41	0.97
	YZ14-402	Hybrid breeding line	YC07-71 × HoCP01-517	15.78	8.5	9.04	0.84
	YZ14-408	Hybrid breeding line	Hocp95-988 × YR05-649	18.66	13.06	11.89	1.54
*Saccharum* ancestry accessions	Badila	*S. officinarum* L	na	20.85	15.2	10.3	0.25
	Guangze bamboo cane	*S.sinense R*	na	18.61	12.34	14.28	—
	Katha	*S.barberi J*	na	19.69	12.13	16.92	—
	India1	*S. spontaneum* L	na	17.21	—	27.47	—
CK	ROC22	Hybrid cultivar	ROC5 × 69–463	20.92	13.07	11.09	1.42

*na* means not available, *HS* means high sucrose content clones, *LS* means low sucrose content clones

### Measuring sucrose content and statistics analysis

Brix (%) was firstly measured by extracting juice from the middle internode of mature stem in the field in Dec. 2016 using a hand refractometer Brix (BX/TDS, ATAGO CO., LTD) as a reference for accession selection for the experiment. The sucrose content was measured by collecting five randomly selected mature stalks of each accession in the field by the second optical rotation test of sucrose in November, December of 2017 and January of 2018 for a total of three times [27]. Meanwhile, the remaining bagasse was soaked in boiling water for 30 min, and then the water was removed, the bagasse was put into a drying oven for drying treatment, after the water was completely evaporated, the fiber content ratio of dry matter weight to fresh weight was calculated for accession. The reduced sugar content was determined by tetramethyl salt volumetric analysis method [28]. The average sucrose content, fiber content and reducing sucrose content data were analyzed by using ANOVA. The significance level (*P-*value) of sucrose difference within and between groups were analyzed by F test in SPSS software.

### DNA extraction, *SPSB* gene amplification, and sequencing

**Fig. 5:**
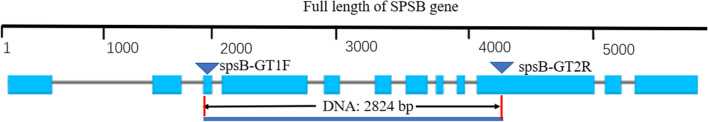
SPSB gene structure and the genomic region amplified by primers, spsB-GR1F and spsB-GT2R. The blue boxes represent exon sequence of SPSB gene, the black lines represent intron sequence, the blue triangle arrows represent primer sites for the amplification. spsB-GT1F: GTGTGCATGGTCTTGTTCGTG; spsB-GT2R: CGCAACCCGTTTCTCCG

The genomic DNA of the 18 accessions was extracted from freshly collected newly emerged leaf samples by using EasyPure® genomic DNA Kit (TransGen Inc.). Primers for *SPS*B amplification were designed by using the Primer5.0 according to the DNA sequence of *SPS*B gene (GenBank ID: JN584485). The forward primer was designed from the third exon and the reverse primer on the 10th exon of *SPS*B gene. The expected length of the amplified fragment was 2,824 bp, containing eight exons and seven introns (Fig. 5), including an important function domain, glycosyl transferase group 1.

The PCR amplification reaction was set by using High-fidelity KOD polymerase (Thermo Scientific Inc., Cat: 11,304,102). PCR products were purified by using EasyPure® PCR purification kit (TransGen Inc., Cat: EP101-02) and then cloned into cloning vectors by using Blunt Zero cloning kit (TransGen Inc., Cat: CB501-01) following the manufacturer’s instructions. For each amplicon from one accession, 60 clones generated from the cloning process were randomly selected for Sanger sequencing in Shanghai Sangon Biotechnology Co., Ltd. (China), and a total of 1080 clones would be generated from 18 accessions.

### Haplotype analyses of *SPSB* gene

The Sanger sequences of each clone were trimmed and assembled by Sequencher (Gene Codes Co., Ann Arbor, MI) with 100% identify and a minimum of six clean nucleotides overlap. At least two or more assemblies with identical sequences were considered as a confirmed haplotype. Multi-alignment of the haplotypes was conducted by using Clustal W [29], and the comparison between haplotypes and *Saccharum spontaneum* AP85-411 genome was performed by BLASTN 2.6.0 + [30]. MEGA 6 software for protein sequence translation and evolutionary analysis [31]. The number of haplotype (Hn), total number of mutations (Eta), number of polymorphic (segregating) sites (S), haplotype diversity (Hd), variance of haplotype diversity (Hv), nucleotide diversity (Pi) were conducted by using DNAsp [32]. The haplotypes from Badila (*S. officinarum*) and India1 (*S. spontaneum*) were used as references for calculating the synonymous (Ks) and non-synonymous (Ka) site by DNAsp. The haplotype network based on the method of Templeton (TCS) was analyzed by using PopART software with default settings [33, 34].

### The correlation analysis of sugar content and variation sites along *SPSB* gene haplotypes

The correlation analysis of sucrose trait and *SPS*B gene haplotypes was conducted by SPSS. The *SPS*B gene haplotype from Badila was set as reference, SNPs and insertion/deletion (InDels) between a haplotype and the reference were represented by arbitrary values,but can distinguish the type of mutation. The transition between C—T is 0.5, A—G is 0.555, the transversion between C—A is 7, C—G is 7.777, T—A is 70, and T—G is 70.777. Insertion was set to 900, and deletion to 900.999 for the correlation analysis. The P-value < 0.01 represents an extremely significant correlation between SNPs (InDels) of haplotype and sucrose content. According to the correlation coefficient matrix, a heat map was drawn to compare the correlation between the different SNPs or InDels of *SPS*B gene haplotype and sucrose, fiber, and reduced sugar content, respectively.

## Supplementary Information

**Additional file 1.** The amplification of SPSB gene for 18 germplasms, M: markers, the size is 5000bp, 3000bp, 2000bp, 1500bp, 1000bp, 750bp, 500bp, 250bp, 100bp;1: Badila, 2: India1, 3: Guangze bamboo cane, 4: katha, 5: YZ02-588, 6: Dezhe93-88, 7: YZ14-401, 8: YZ14-405, 9: YT00-236, 10: YZ14-407, 11: GT12, 12: YZ94-343, 13: YZ14-402, 14: YZ14-403, 15: YZ14-404, 16: YZ14-406, 17: YZ14-408, 18: ROC22.

**Additional file 2.** The comparative information between the 39 haplotypes of *SPS*B gene and the reference sequence of AP85-411 genome.

**Additional file 3.** Variation sites of single nucleotide polymorphism (SNP) and InDels in the 39 *SPS*B haplotypes.

**Additional file 4.** The sequence variations of positive selection haplotype in clones of high sucrose content (HS) and low sucrose content (LS).

## Data Availability

The dataset of the 39 haplotype sequences supporting the conclusions of this article is available in the National Center of Biotechnology Information (NCBI) GenBank https://www.ncbi.nlm.nih.gov/ with accession numbers of OP615365 ~ OP615403. The rest intermediate analysis data and the plant materials are available upon request from the first author.

## Abbreviations

YAAS: Yunnan Academy of Agricultural Sciences, China
YSRI: Sugarcane Research Institute of Yunnan
NNSGR: National Nursery of Sugarcane Germplasm Resources, China
SNP: Single nucleotide polymorphism
InDels: Insert and delete sequence
Ka: Non-synonymous substitution
Ks: Synonymous
Hn: The number of haplotype
Eta: Total number of mutations
S: Number of polymorphic (segregating) sites
Hd: Haplotype diversity
Hv: Variance of haplotype diversity
Pi: Nucleotide diversity
TCS: The method of Templeton, Crandall and Sing
ANOVA: Analysis of Variance
HAP: Haplotype
SPS: Sucrose phosphate synthase
HS: High sucrose content accession
LS: Low sucrose content accession
BACs: Bacterial artificial chromosomes
UDPG: Uridine diphosphate glucose
Sq: Chi-square
Df: Degrees of freedom
Mse: Mean Squared Error
