# Supplementary figures and images for: Haplotype variations of sucrose phosphate synthase B gene among sugarcane accessions with different sucrose content
Source: BMC Genomics. 2023 Jan 25;24:42. doi: 10.1186/s12864-023-09139-1 (PMC9875459; doi:10.1186/s12864-023-09139-1)

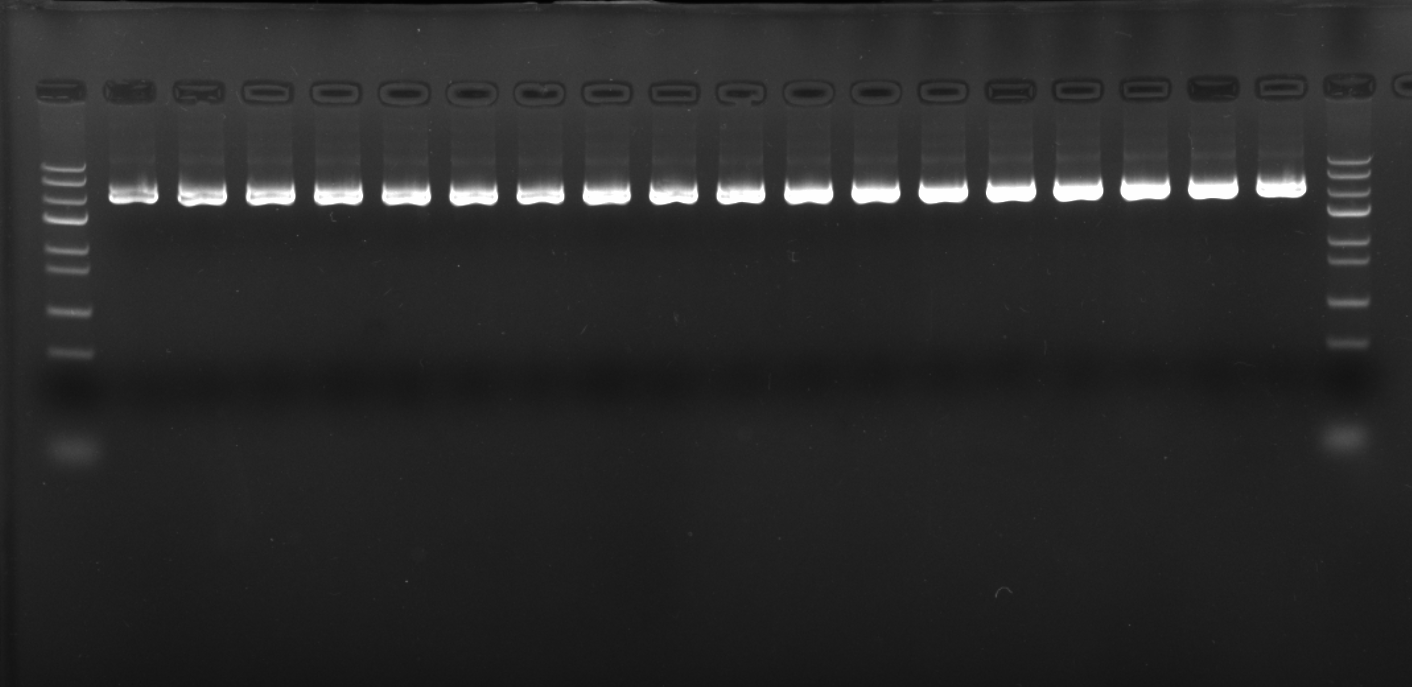


M 1 2 3 4 5 6 7 8 9 10 11 12 13 14 15 16 17 18 M

Supplement: Supplementary file 1 — Additional file 1. The amplification of SPSB gene for 18 germplasms, M: markers, the size is 5000bp, 3000bp, 2000bp, 1500bp, 1000bp, 750bp, 500bp, 250bp, 100bp;1: Badila, 2: India1, 3: Guangze bamboo cane, 4: katha, 5: YZ02-588, 6: Dezhe93-88, 7: YZ14-401, 8: YZ14-405, 9: YT00-236, 10: YZ14-407, 11: GT12, 12: YZ94-343, 13: YZ14-402, 14: YZ14-403, 15: YZ14-404, 16: YZ14-406, 17: YZ14-408, 18: ROC22. [file 12864_2023_9139_MOESM1_ESM.docx]

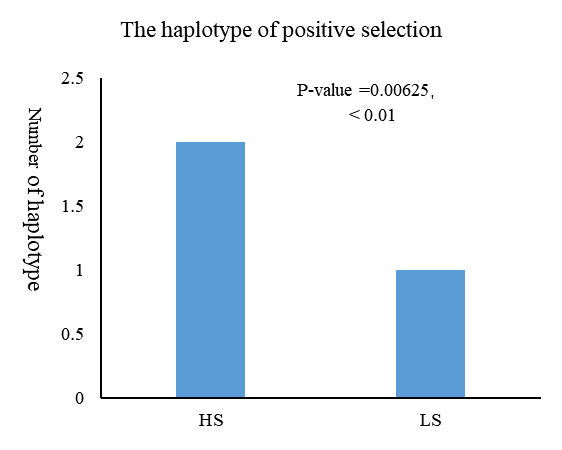

Supplement: Supplementary file 4 — Additional file 4. The sequence variations of positive selection haplotype in clones of high sucrose content (HS) and low sucrose content (LS). [file 12864_2023_9139_MOESM4_ESM.doc]
